# Development of an Animal-Assisted Activity/Therapy Dog Checklist for Long-Term Care Facilities

**DOI:** 10.1093/geroni/igab046.3099

**Published:** 2021-12-17

**Authors:** Karen Dunn, Amy Johnson, Melissa Winkle

**Affiliations:** 1 Oakland University, Rochester, Michigan, United States; 2 American Occupational Therapy Organization, North Bethesda, Maryland, United States

## Abstract

Animal-assisted activities (AAA) and therapy standards of practice have been published to protect the well-being of animals, animal handlers, and the special populations of patients that benefit from this mode of treatment. Inconsistencies among practice standards with concerns surrounding the topics of dog welfare, human well-being, and zoonotic transmission have been reported. The purpose of this qualitative research study was to review published AAA and therapy standards with older adult populations for best practices, conduct focus group sessions with caregivers from long-term care facilities that allow therapy dog visitation, and synthesize findings into an AAA checklist to be used by long-term care facility decision-makers when interviewing or bringing in therapy dog teams. Comparative analyses utilizing a systematic and sequential approach was used to analyze the data from the focus group sessions. Due to the COVID-19 pandemic, only two focus group sessions at one long-term care facility were conducted resulting in a total of 15 caregivers. Four themes emerged from the data: promotes positive mood, essential resident screenings, caregiver roles, and memory aides. Relevant themes and AAA and therapy standards and guidelines were then combined in the development of the AAA/Therapy Dog Checklist. Administrators may find having a user-friendly AAA/therapy dog checklist a useful tool that can be used when interviewing therapy dog teams to ensure future dog therapy experiences will be positive and safe. The safety and well-being of residents in long-term care facilities and animals are essential to promote positive health outcomes for both populations.

